# CMR assessment of right ventricular function in patients with combined pulmonary stenosis and insufficiency after correction of tetralogy of Fallot

**DOI:** 10.1186/1532-429X-11-S1-O54

**Published:** 2009-01-28

**Authors:** Maureen Kohi, Karen G Ordovas, Charles B Higgins

**Affiliations:** University of California, San Francisco, San Francisco, CA USA

**Keywords:** Right Ventricular, Pulmonary Stenosis, Right Ventricular Function, Right Ventricular Ejection Fraction, Pulmonary Regurgitation

## Introduction

Tetralogy of Fallot is the most common form of cyanotic congenital heart disease. Following surgical correction, most patients present with pulmonary insufficiency (PI) which plays a pivotal role in right ventricular (RV) dysfunction. Multiple prior studies have demonstrated the accuracy of CMR for assessment of RV volumes, ejection fraction and pulmonary regurgitant fraction, especially in the setting of an enlarged right ventricle. The deleterious effect of chronic pulmonary regurgitation on RV function is well known and pulmonary valve replacement has been shown to improve ventricular function in these patients. However, the effects of residual pulmonary stenosis on RV function are not well understood. Using CMR and conductance catheter techniques, a study performed in a growing pig model demonstrated that chronic pulmonary stenosis and insufficiency (PSPI) results in improved RV myocardial contractility when compared to a group with isolated PI, indicating a possible protective effect. The combined effect of PSPI on RV function has never been reported in a human model.

## Purpose

The purpose of this study was to compare CMR parameters for assessment of RV volumes and function between patients with combined PSPI and isolated PI following surgical repair of Tetralogy of Fallot.

## Methods

A retrospective review of patients with corrected Tetralogy of Fallot who had undergone CMR and echocardiogram was performed. A total of 46 patients were included: 9 patients with PSPI and 37 patients with isolated PI. Cine MRI images in the short-axis plane were used to calculate the following parameters: pulmonary regurgitant fraction (PRF), RV end-diastolic volume (RVEDV), RV end-systolic volume (RVESV), RV stroke volume (RVSV) and RV ejection fraction (RVEF). RV end-diastolic volume and end-systolic volume indexes (RVEDVi, RVESVi) were calculated based on the body surface area. Peak pressure gradient across the pulmonary valve was obtained from echocardiogram performed within 3 months of the CMR. Means and standard deviations of the CMR parameters were compared between the combined PSPI and isolated PI groups using Student's t-test. A p < 0.05 was considered statistically significant.

## Results

RVEF was significantly higher in combined PSPI patients (47 ± 9%) than in isolated PI patients (40 ± 10%) (p = 0.042). RVESVi was significantly lower in combined PSPI patients (67.3 ± 19 ml/m^2^) than in patients with isolated PI (92.1 ± 43 ml/m^2^) (p = 0.026). There was no significant difference between RVEDVi (p = 0.44) and PRF (P = 0.38) in the two groups.

## Conclusion

RV function as assessed by RVEF and RVESVi was improved in patients with combined PSPI when compared to patients with isolated PI following surgical correction of Tetralogy of Fallot with similar degree of pulmonary regurgitation.

